# Passive Wireless Hermetic Environment Monitoring System for Spray Painting Workshop

**DOI:** 10.3390/s16081207

**Published:** 2016-08-01

**Authors:** Lifeng Wang, Jingjing Ma, Yan Huang, Dan Tang, Qing-An Huang

**Affiliations:** 1 Key Laboratory of MEMS of the Ministry of Education, Southeast University, Nanjing 210096, China; woshihuangyansan@163.com (Y.H.); savage9@126.com (D.T.); hqa@seu.edu.cn (Q.-A.H.); 2 Smart Integrated Sensor Engineering Center, Jiangsu R & D Center for Internet of Things, Wuxi 214135, China; majingjing@ciotc.org

**Keywords:** passive wireless, environment monitoring, inductive coupling, spray painting workshop

## Abstract

Passive wireless sensors have the advantages of operating without a power supply and remote sensing capability. Hence, they are very suitable for some harsh environments, such as hermetic environments, rotating parts, or very high temperature environments. The spray painting workshop is such a harsh environment, containing a large amount of flammable paint mist and organic gas. Aiming at this special environment of spray painting workshop, a passive wireless hermetic environment monitoring system was designed, fabricated, and demonstrated. The proposed system is composed of a transponder and a reader, and the circuit design of each part is given in detail in this paper. The power and the data transmission between the transponder and the reader are realized by the inductive coupling mechanism. Utilizing the back scatter modulation and channel multiplexing, the frequency signals generated by three different environmental sensors—together with their interfaces in the transponder—are wirelessly read out by the reader. Because of the harsh environment of the spray painting room, the package of the monitoring system is quite important. Three different kinds of filter films for the system package were compared. The experimental results show that the composite filter film aluminum anodic oxide/polytetrafluoroethylene (AAO/PTFE) has the best performance. After fabrication, the measured temperature, humidity, and pressure sensitivities were measured and found to be 180 Hz/°C in the range of 0~60 °C, 100 Hz/%RH in the range of 15~95 %RH, and 42 Hz/hPa in the range of 600~1100 hPa, respectively. Additionally, the remote sensing distance of the monitoring system reaches 4 cm. Finally, the passive wireless hermetic environment monitoring system was installed on the glass wall of the spray painting workshop and was successfully demonstrated.

## 1. Introduction

The sensor technology has penetrated into industry and daily life in all aspects [1]. However, traditional sensors are not easy, or even unable, to measure some harsh environments, such as sealed environments, rotating parts, or very high temperature environments. The reason for this is that traditional sensors are active powered and/or wire connected. Aiming at these shortcomings of traditional sensors, passive wireless sensors have been invented and applied to these special environments [2,3,4]. The most widely used mechanism is inductive coupling [5].

The first passive wireless sensor was reported by Collins in 1967 [6], which measures intraocular pressure with a pair of flat coils. However, for a long time after this period, passive wireless sensors had not been given much attention. Until 1996, a totally integrated Inductor–capacitor (LC) type passive wireless intraocular pressure sensor was proposed by Puers et al. [7]. After 2000, with the development of the micromachining process, passive wireless sensing technology began to develop quickly. Besides non-contact pressure detection, passive wireless sensing technology has been used to measure many other parameters [8,9,10,11]. A passive wireless humidity sensor which could be used to monitor the moisture in sealed package was proposed by Zhang at al. [12]. Using ferroelectric ceramic as the temperature sensing material, a passive wireless temperature sensor is reported in [13], which can work in environments with temperatures up to 700 °C. In some cases, several parameters need to be monitored at the same time. Using several resonators with different resonant frequencies or one resonator with multi-resonant frequencies, passive wireless multi-parameter sensing could be achieved [14,15,16]. Besides, adding back-scatter modulation together with channel multiplexing into the inductive coupling system, a passive wireless multi-parameter sensing system could be easily realized [17,18]. 

In this study, passive wireless multi-parameter sensing technology was used to monitor the environmental parameters of a spray painting workshop. In order to ensure the quality of spraying and the safety of the working environment, the environmental parameters—including the temperature, humidity, and pressure—of a spray painting workshop need to be monitored in real time. For example, temperature affects the rate of solvent evaporation. However, the air in the spray painting workshop contains a large amount of flammable paint mist and organic gas, so wire-powered devices are forbidden; even the lamps used for lighting are all installed outside the glass wall of the spray painting workshop. Hence, the passive wireless sensing technology is very suitable for this situation, which has the advantages of operating without a power supply and remote sensing capability. In Section 2, the overall block diagram and the working principle of the system is introduced. Then, the circuit design of each part is given in detail. Because of the harsh environment of the spray painting room, the package of the monitoring system is quite important. Section 3 describes the package of the monitoring system. The measurement and demonstration of the system are shown in Section 4. Finally, some conclusions are given in Section 5.

## 2. Circuits Design

### 2.1. Overall Block Diagram

**Figure 1:**
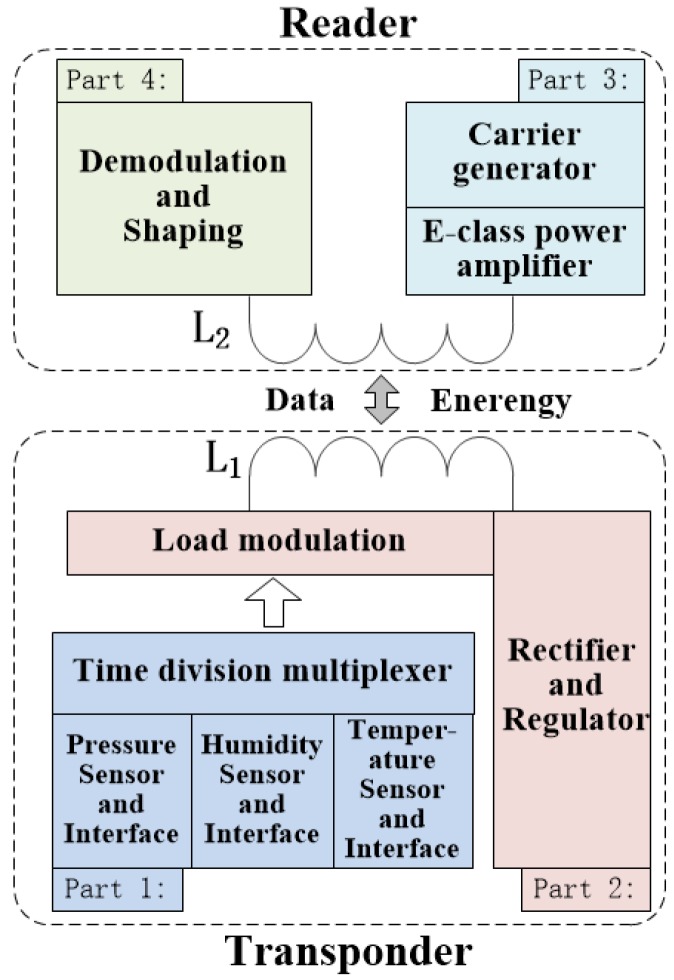
Overall block diagram of the passive wireless hermetic environment monitoring system. The system is composed of a reader and a transponder. The power and the data transmission between the transponder and the reader are realized by way of inductive coupling.

The passive wireless hermetic environment monitoring system for the spray painting workshop is composed of a transponder and a reader; the overall block diagram of the system is shown in Figure 1. The power and the data transmission between the transponder and the reader are realized by way of inductive coupling. Hence, the transponder can be used to monitor hermetic environments without wires or batteries. 

The working process of the system is as follows. Firstly, the carrier generator together with the E-class power amplifier in the reader generates a carrier signal. Then, the carrier signal in the reader is coupled to the transponder though their inductors. The coupled carrier signal in the transponder is then rectified and regulated as the voltage source for itself. With sufficient power supply, the temperature, humidity, and the pressure sensors together with their interfaces in the transponder can each generate a digital frequency signal, whose value is decided based on the environment. In order to realize the monitoring of multiple environmental parameters, the time division multiplexer is used to sequentially transmit three digital frequency signals. Then, the output signals of the time division multiplexer are modulated to the carrier signal by the load modulation circuit in the transponder, and the modulated carrier signal is, at the same time, coupled back to the reader. Finally, the digital frequency signals generated by the sensors and their interfaces are recovered from the modulated carrier signal by the demodulation and shaping circuits. The detail designs of the transponder and the reader are given below.

### 2.2. Design of the Transponder

#### 2.2.1. Part 1: Sensor Interface Circuits and Time Division Multiplexer

The function of the sensor interface circuit is to convert the capacitive sensor signal to a digital frequency signal. A lot of circuits can convert capacitive variations to frequency signals. Here, we used the multi-harmonic oscillator, because of its low temperature drift characterization.

**Figure 2:**
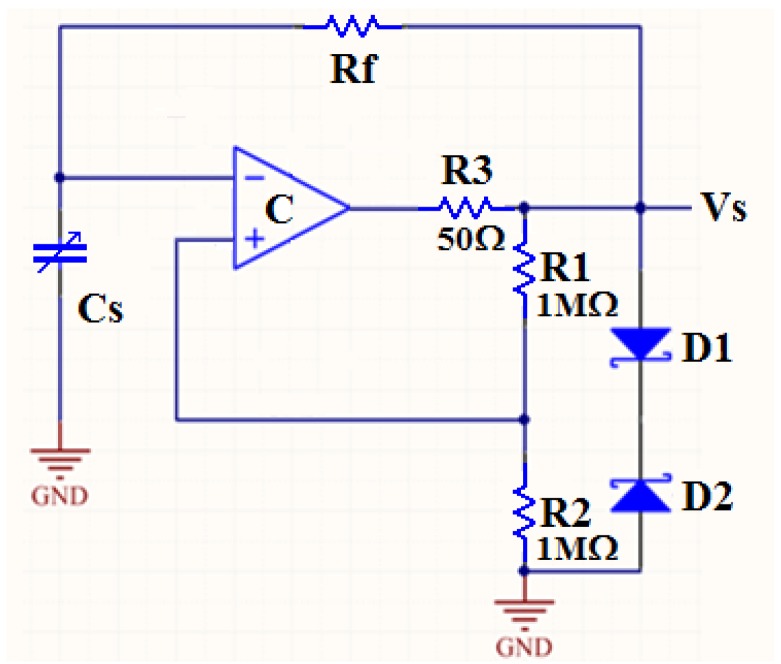
Schematic of the sensor interface circuit. By this multi harmonic oscillator, the capacitor C_s_ could be converted to frequency signal V_s_.

Figure 2 shows the schematic of the multi-harmonic oscillator. In Figure 2, the capacitor C_s_ represents a capacitive sensor. During the circuit design process, we use variable capacitors to represent capacitive sensors. The resistor R_f_ and the op amp constitute an integral circuit, which plays the role of frequency selection. The resistance R_1_ and R_2_ constitute the positive feedback circuit, and the diode D_1_ and D_2_ have the function of bidirectional limiting of voltage. The output frequency *f* of the multi-harmonic oscillator can be calculated by [19]:

(1) $$f=\frac{1}{2R_{f}C_{s}ln(1+\frac{2R_{2}}{R_{1}})}$$

**Figure 3:**
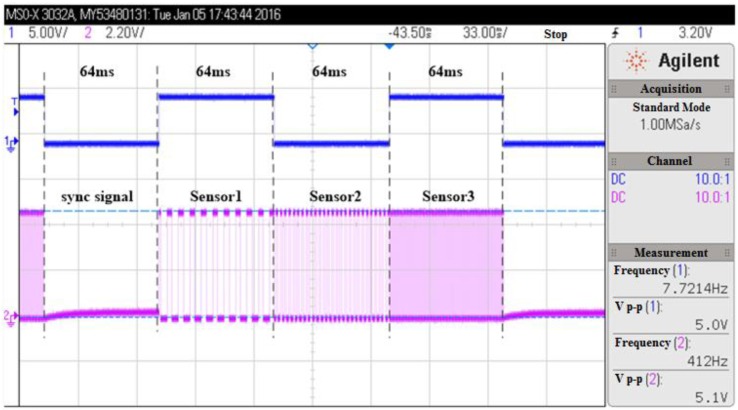
Output signals of the time division multiplexing circuit. The sync signal and the signals of three sensors are sequentially outputted. The transmission time for each signal is designed to be 64 ms.

There are three kinds of environmental sensors integrated in the transponder, but the monitoring system has only one data transmission channel. Therefore, the time division multiplexer is used to realize the time division multiplexing transmission. The time division multiplexer consists of the oscillator, the frequency divider and the multiplexer. Figure 3 shows the output signals of the time division multiplexer. It can be seen that the sync signal and the signals of three sensors are sequentially outputted, and the transmission time for each signal was designed to be 64 ms.

#### 2.2.2. Part 2: Rectifier, Regulator, and Load Modulation Circuit

**Figure 4:**
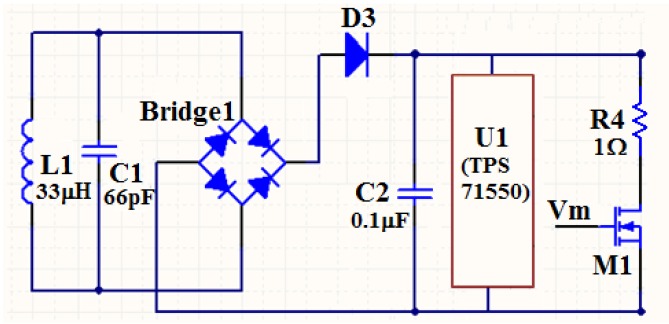
Schematic of the rectifier, regulator, and load modulation circuit. L_1_ is the planar inductor that receives the carrier signal from the reader. The diode bridge plays the role of rectifier. The diode D_3_, together with the capacitor C_2_, filters the signal after rectification. The regulator chip U_1_ (TPS71550) is used to make the dc voltage more smooth and stable. The NMOS transistor M1 as well as the resistor R4 forms the load modulation circuit.

The rectifier together with the regulator converts the received ac carrier signal to a stable dc voltage, which is used as the power supply for the transponder itself. The load modulation circuit modulates the low frequency data signals from the time division multiplexer to the high frequency carrier signal. The schematic of the rectifier, regulator, and load modulation circuit is shown in Figure 4. L_1_ is the planar inductor that receives the carrier signal from the reader. The diode bridge plays the role of rectifier. The diode D_3_, together with the capacitor C_2_, filters the signal after rectification. Finally, a regulator chip U_1_, TPS71550 [20], is used to make the dc voltage more smooth and stable.

The load modulation circuit consists of the NMOS transistor M_1_ and the resistor R_4_. The mechanism of the load modulation can be simply explained as follows. The low frequency signal V_m_ changes the on/off state of M_1_, so the equivalent load impedance in the reader that coupled from the transponder synchronously changes, and the change of the load impedance of the reader finally leads to the amplitude variation of the carrier signal. The result is that the input low frequency signal V_m_ is modulated to the high frequency carrier signal. Hence, the load modulation we used here actually belongs to the AM modulation. For better recovery of the sensor data, the frequency of the sensor signal must be at least one order of magnitude lower than the carrier signal [21]. Besides, a large modulation depth is also very helpful, which can be acquired from the large impedance variation of the load modulation circuit and the high Q factors of the transponder inductor and the reader inductor.

**Figure 5:**
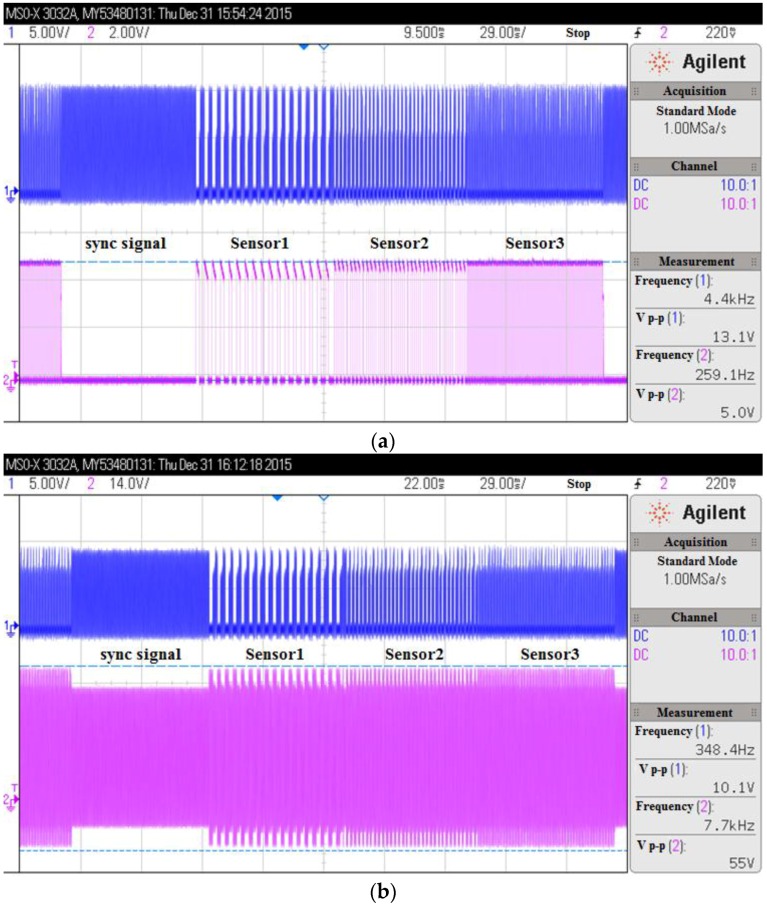
The signal waves of the load modulation circuit. (**a**) The bottom wave is the unmodulated sensor signals from the time division multiplexer, the top wave is the modulated carrier signal at the transponder inductor; (**b**) The bottom wave is the modulated signals at the transponder inductor, and the top wave is the modulated carrier signal at the reader inductor.

Figure 5 shows the signal waves of the load modulation circuit. In Figure 5a, the bottom wave is the unmodulated sensor signals from the time division multiplexer, the top wave is the modulated carrier signal at the transponder inductor. In Figure 5b, the bottom wave is the modulated signals at the transponder inductor, and the top wave is the modulated carrier signal at the reader inductor. It can be seen that the sync signal and the three sensors’ signals are successfully modulated to the carrier signal. The frequency of the carrier signal in our system is 5.1 MHz.

### 2.3 Design of the Reader

#### 2.3.1. Part 3: Carrier Generator and E-Class Power Amplifier

**Figure 6:**
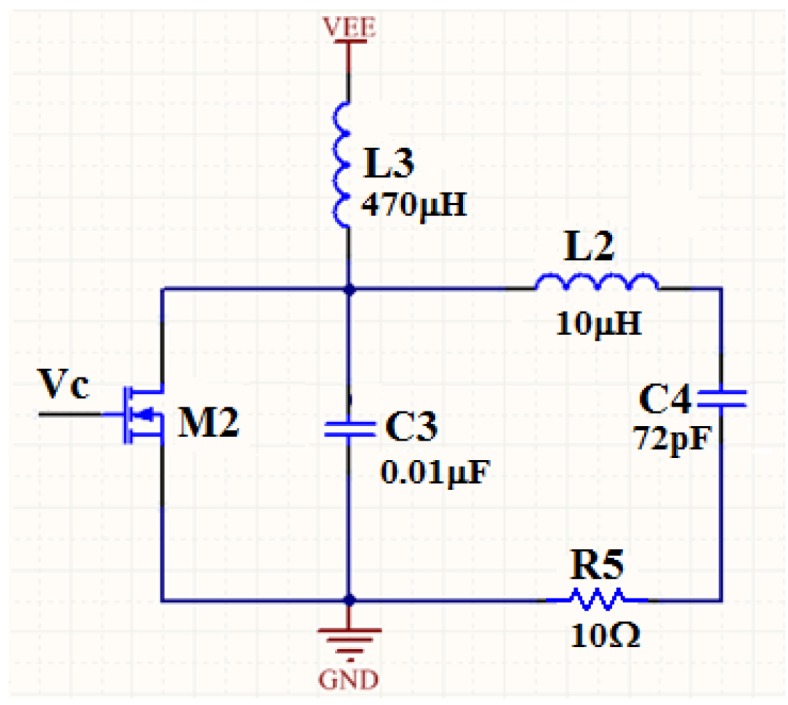
Schematic of the E-class power amplifier. When the LCR branch (L_2_, C_4_, and R_5_) is on its resonant state, the amplitude of the output carrier signal will reach the maximum value.

In our passive wireless sensing system, the carrier signal not only transmits sensor data but also provides power for the transponder. Therefore, a carrier signal with large amplitude is needed. Firstly, an oscillator chip LTC6900 [22] is used to generate a 50% duty cycle square wave. The output frequency of LTC6900 is adjustable from 1 kHz to 20 MHz, which is high enough for our system. However, the amplitude of the output wave is less than 5 V, which is too weak to power the transponder. Hence, the output of the oscillator chip needs to be amplified. The E-class power amplifier, which has very high efficiency, is used here to amplify the output wave of the oscillator chip. Figure 6 shows the schematic of the E-class power amplifier. V_c_ is the output wave from the oscillator chip. If the capacitance of C_3_ and (in parallel) the MOS transistor M_2_ and the parameters of the LCR branch (L_2_, C_4_, and R_5_) are regulated just right, the LCR branch will be on its resonant state and the carrier amplitude on inductor L_2_ will reach the maximum value. When the LCR branch is on its resonant state, the amplitude of the output carrier signal will be decided by the dc power supply of the E-class amplifier. The output amplitude of the carrier signal reaches 63 V when the dc power supply is set to 12 V.

#### 2.3.2. Part 4: Demodulation and Shaping Circuits

**Figure 7:**
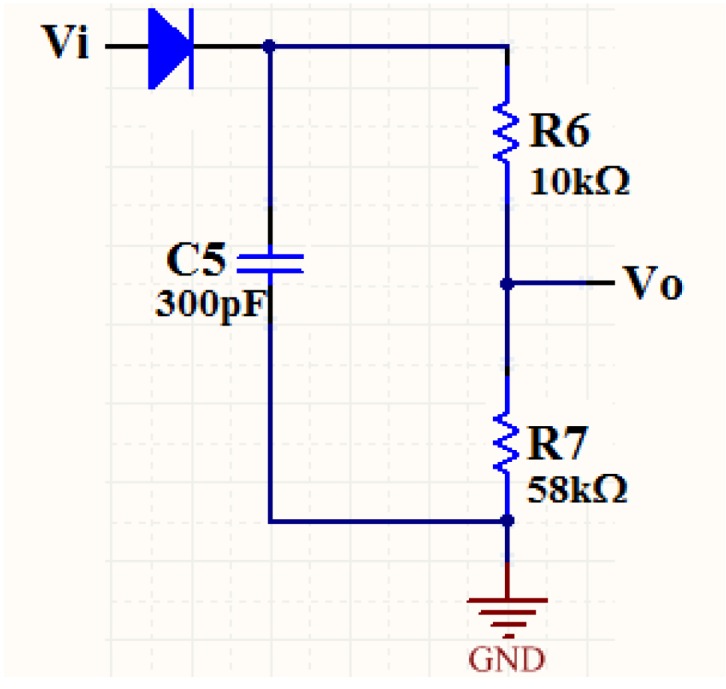
Schematic of the demodulation circuit. The envelope detection of the input V_i_ is realized by the diode, which has the property of unidirectional conductivity.

After the reader inductor receives the modulated signal from the transponder inductor, demodulation and shaping circuits are needed to recover the sensor data. The demodulation circuit is realized by using a diode together with a capacitor and two resistors. The schematic of the demodulation circuit is shown in Figure 7. The diode has the property of unidirectional conductivity, so the capacitor C_5_ will be charged if the input signal V_i_ is positive and will discharge if V_i_ is negative. The charging and the discharging processes are carried out repeatedly and alternatively, which realize the envelope detection of the input V_i_. Appropriate value selection of the resistors and the capacitor can minimize the distortion of the demodulated signal.

**Figure 8:**
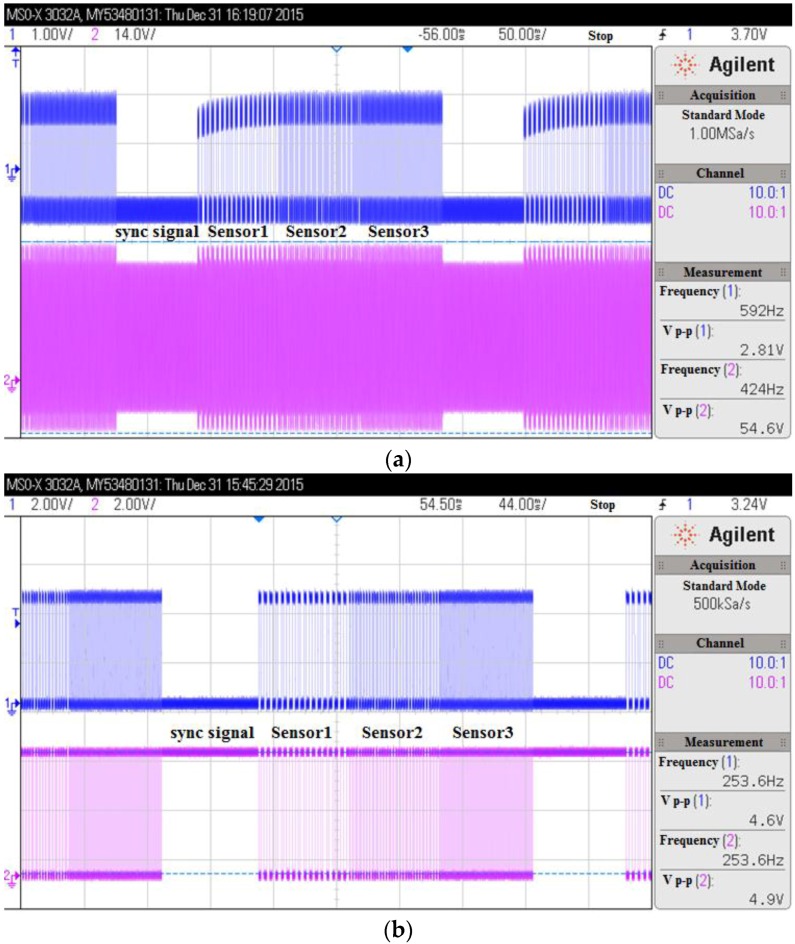
(**a**) The input modulated signal and the output demodulated signal of the demodulation circuit: the signal wave below is the modulated signal, and the signal wave above is the demodulated signal; (**b**) The signals of the shaping circuit: the signal wave above is the output of the voltage follower, and the signal wave below is the final output signal from the hysteresis comparator.

Figure 8a shows the input modulated signal and the output demodulated signal of the demodulation circuit: the signal wave below is the modulated signal and the signal wave above is the demodulated signal. It can be seen that the demodulated signal has a relatively small amplitude, and, at the same time, a lot of glitches. So the shaping circuit, which contains the voltage follower and the hysteresis comparator, is necessary. Figure 8b shows the signals of the shaping circuit: the signal wave above is the output of the voltage follower and the signal wave below is the final output signal from the hysteresis comparator. It can be seen that through the inductive coupling based back-scatter modulation, diode based demodulation, and the shaping process, the sensor data generated by the transponder is finally outputted from the reader.

**Figure 9:**
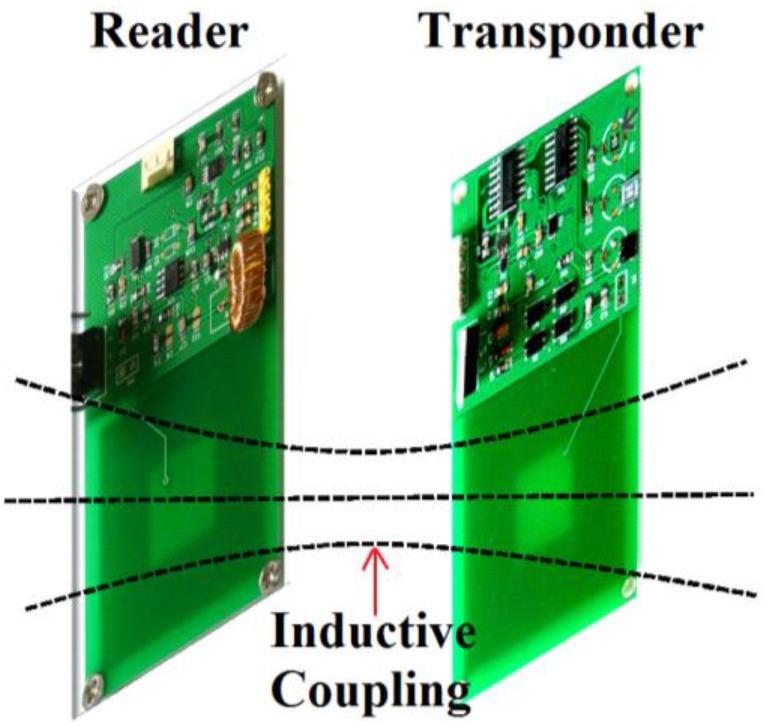
Photograph of the reader and the transponder. The sizes of the inductors of the reader and the transponder are both 4 cm × 4 cm. The inductive coupling between these two inductors realizes the passive wireless sensing.

Figure 9 shows the photograph of the fabricated reader and the transponder. The area of the reader and transponder are both 5 cm × 8.8 cm, and the sizes of the inductors of the reader and the transponder are both 4 cm × 4 cm. Three environment monitoring sensors, including the capacitive temperature sensor, the capacitive humidity sensor, and the capacitive pressure sensor, are mounted on the transponder. Both the temperature and humidity sensors are the capacitive structures filled with corresponding sensitive dielectric materials. More details can be found in our previous works [15,23]. The capacitive pressure sensor is a commercial product, SCB10H from VTI [24], in which the pressure sensing mechanism is based on the bending of a silicon diagram.

## 3. Package of the Transponder

In a spray painting workshop, two kinds of contaminants are produced during the painting process: the paint mist and the organic gas. Among them, the paint mist is the main factor that leads to the sensor failure. The diameter of most paint mists are less than 10 μm, and they are very easy to adhere to objects. If no package is mounted, the paint mists will deposit on sensor chips and quickly lead to sensor failure.

**Figure 10:**
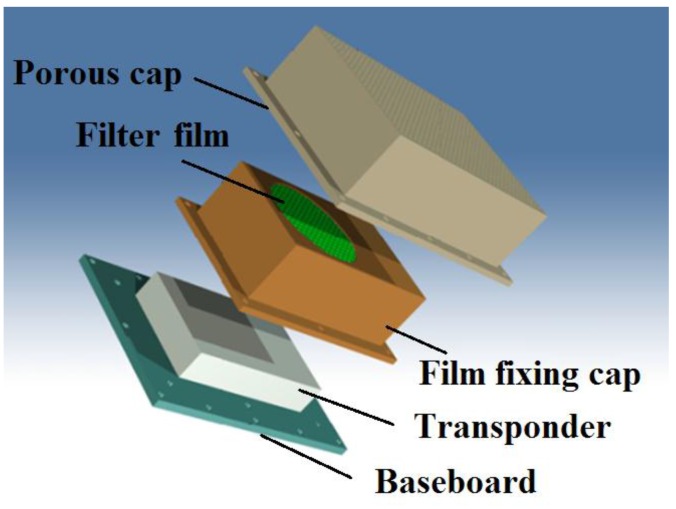
Schematic of the package of the transponder. Firstly, the transponder is mounted on the baseboard. Then, the transponder is covered with the film fixing cap. The filter film on the film fixing cap is used for resisting paint mist. Finally, the film fixing cap is covered with the porous cap, which is used for blocking dust and large particles.

However, the hermetically sealed package is not suitable for the transponder, which includes the humidity sensor and the pressure sensor. In the circumstance of the spray painting workshop, the package of the transponder should not only be permeable to air but also be resistant to the deposition of the paint mist. Figure 10 shows the schematic of the package of the transponder. Firstly, the transponder is mounted on the baseboard. Then, the transponder is covered with the film fixing cap. The filter film on the film fixing cap is used for resisting paint mist. Finally, the film fixing cap is covered with the porous cap, which is used for blocking dust and large particles. 

**Figure 11:**
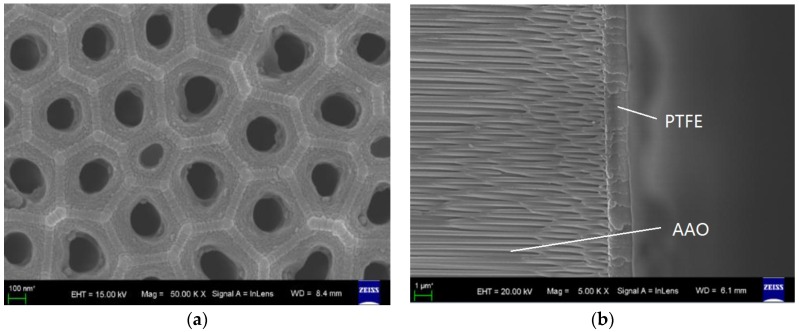
SEM photographs of the aluminum anodic oxide/polytetrafluoroethylene (AAO/PTFE) composite film (**a**) the surface view; (**b**) the section view.

Therefore, the filter film plays the most important role in the package of the transponder. Based on the environment of the spray painting workshop, we designed a composite film that is composed of a porous aluminum anodic oxide (AAO) base film and a polymeric polytetrafluoroethylene (PTFE) film. The AAO base film filters chemical macromolecules or impurity particles in the air, and provides flow channels for air. The polymeric PTFE film is used to obtain a hydrophobic surface, in other words, to prevent paint mist adhesion. The fabrication of the AAO/PTFE composite film is not very complex. Firstly, the surface oxide film of the prepared aluminum wafer is removed by alkali solution. Secondly, the anodic oxidation process is performed for the first time and then the oxide film is also removed. Thirdly, the anodic oxidation process is performed for the second time, followed by the hole enlarging treatment and heat treatment. Fourthly, using the spin coating process, a hydrophobic and microporous PTFE membrane is coated on the AAO film. Finally, the PTFE film is hardened in vacuum constant temperature drying oven. The SEM photographs of the surface and the section view of the AAO/PTFE composite film are shown in Figure 11. In Figure 11a, the average diameter of the holes is about 200 nm. Figure 11b shows that the AAO film is covered with the PTFE layer, and the PTFE material already seeps into the holes of the AAO film. The thicknesses of the AAO film and the PTFE layer are about 10 μm and 1.5 μm, respectively.

**Figure 12:**
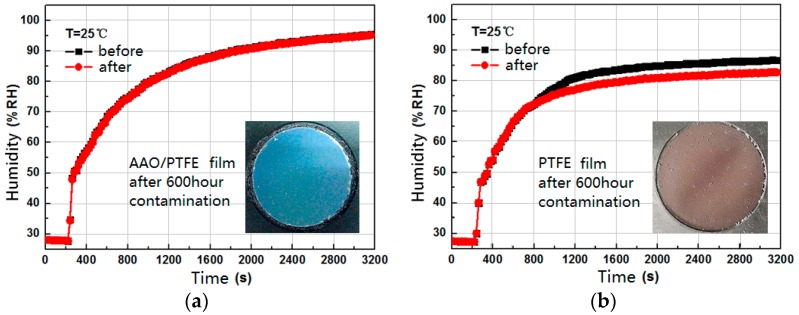

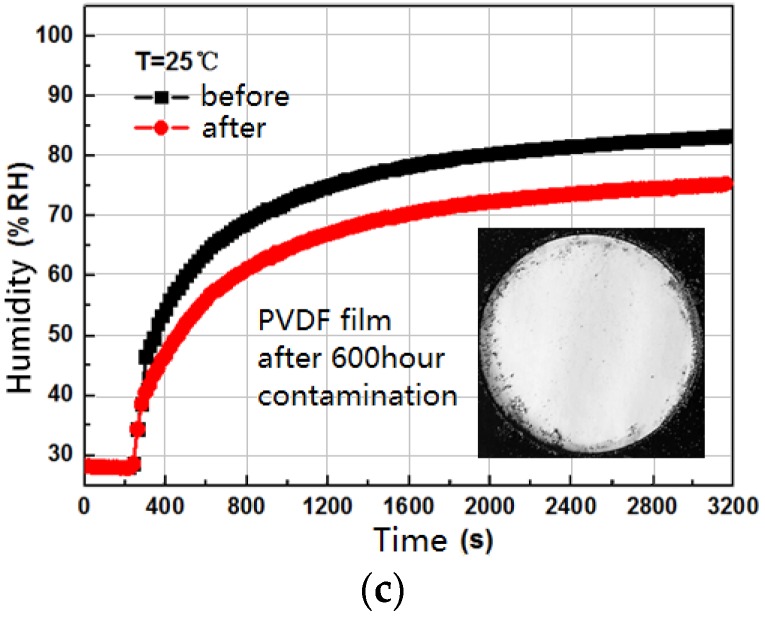
Transient responses of the humidity sensors covered by different filter films before and after 600 h paint mist contamination, (**a**) AAO/PTFE film; (**b**) PTFE film; and (**c**) polyvinylidene fluoride (PVDF) film.

A contrast experiment was carried out to compare the performance of the fabricated composite filter film with two other kinds of filter films, PTFE film and polyvinylidene fluoride (PVDF) film. The PTFE and PVDF films are both large-scale commercial polymeric materials. They have good air permeability, good corrosion resistance, and low surface tension. Other films that have similar properties as above, such as polypropylene films [25] and Nafion films [26], may be possible candidates. Here, we mainly investigated the change of water vapor flux and the antipollution properties of each filter film after being exposed for a long time to pollution in the spray painting workshop. Figure 12 gives the transient responses of the humidity sensors before and after 600 h paint mist contamination; the sensors were covered by the AAO/PTFE film, the PTFE film, and the PVDF film, respectively. The photographs of the polluted films are also shown in Figure 12. It can be seen that, after a long time period of working in the spray painting workshop, the composite filter film AAO/PTFE has the minimum pollution and the smallest change of water vapor flux.

## 4. Measurement and Demonstration

**Figure 13:**
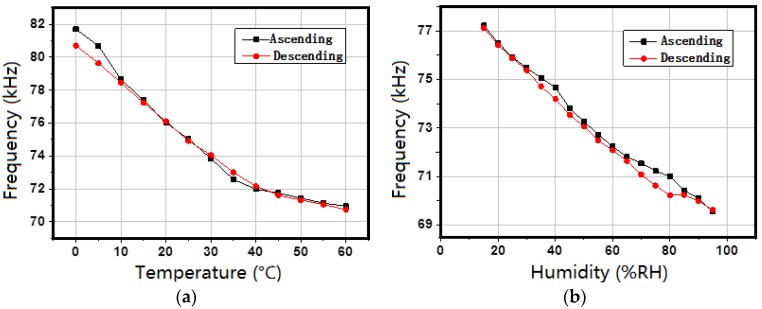

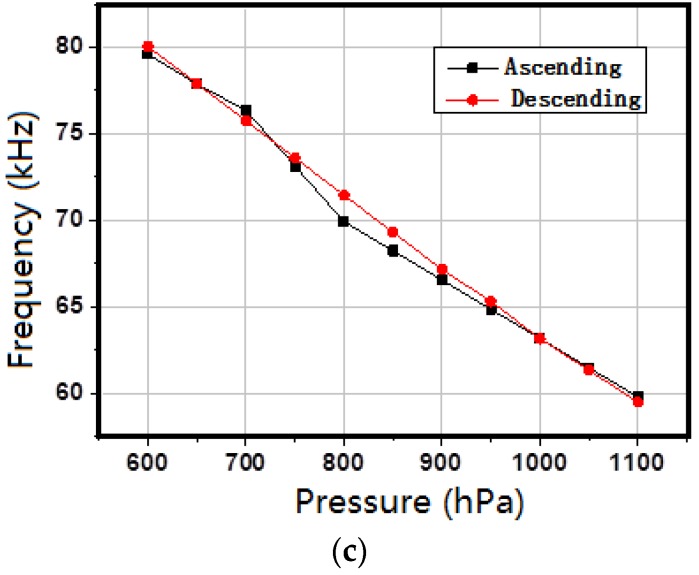
Output frequency versus (**a**) temperature; (**b**) humidity; (**c**) pressure.

By using the OMEGA205 C340 temperature and humidity chamber (made by Vötsch Industrietechnik), the temperature and humidity characterizations of the passive wireless monitoring system were measured. In Figure 13a, the variation of the output frequency is about 11 kHz when the temperature changes from 0 °C to 60 °C. Hence, the average temperature sensitivity of the monitoring system is 180 Hz/°C. Figure 13b shows that the output frequency changes about 8 kHz when the humidity varies from 15 %RH to 95 %RH, and the humidity sensitivity can be calculated as 100 Hz/%RH.

The pressure characterization of the passive wireless monitoring system was measured by the GY-600 pressure chamber (made by XiangHe, GuoRuiZhi Test Equipment Co., Ltd., Beijing, China). The measured relationship between the pressure and the output frequency is shown in Figure 13c. The variation of the output frequency is about 21 kHz when the pressure changes from 600 hPa to 1100 hPa. So the pressure sensitivity of the monitoring system is 42 Hz/hPa.

**Figure 14:**
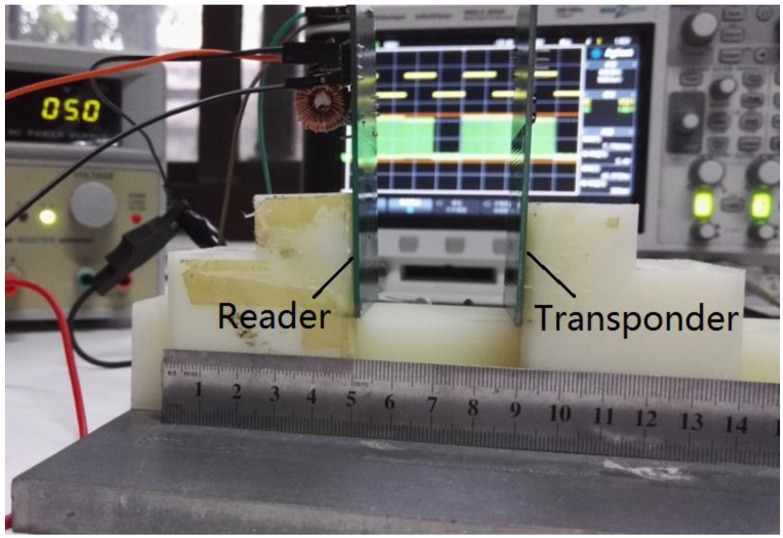
Measurement of passive wireless transmission distance. This ruler shows the passive wireless transmission distance between the transponder and the reader reaches 4 cm.

The passive wireless transmission distance of the monitoring system was also measured. The test platform as well as the measurement result is shown in Figure 14. When the distance between the transponder and the reader reaches 4 cm, the output signal of the reader is still working properly. Under fixed inductor size and input power, adjusting the resonant frequency of the transponder and the reader to the carrier signal frequency could be an efficient way to increase the telemetry distance. Because the wall of the spray painting workshop is made of glass, the effect of glass in the distance measurement needs to be considered. However, the measurement result shows that glass has no influence on the passive wireless sensing distance.

**Figure 15:**
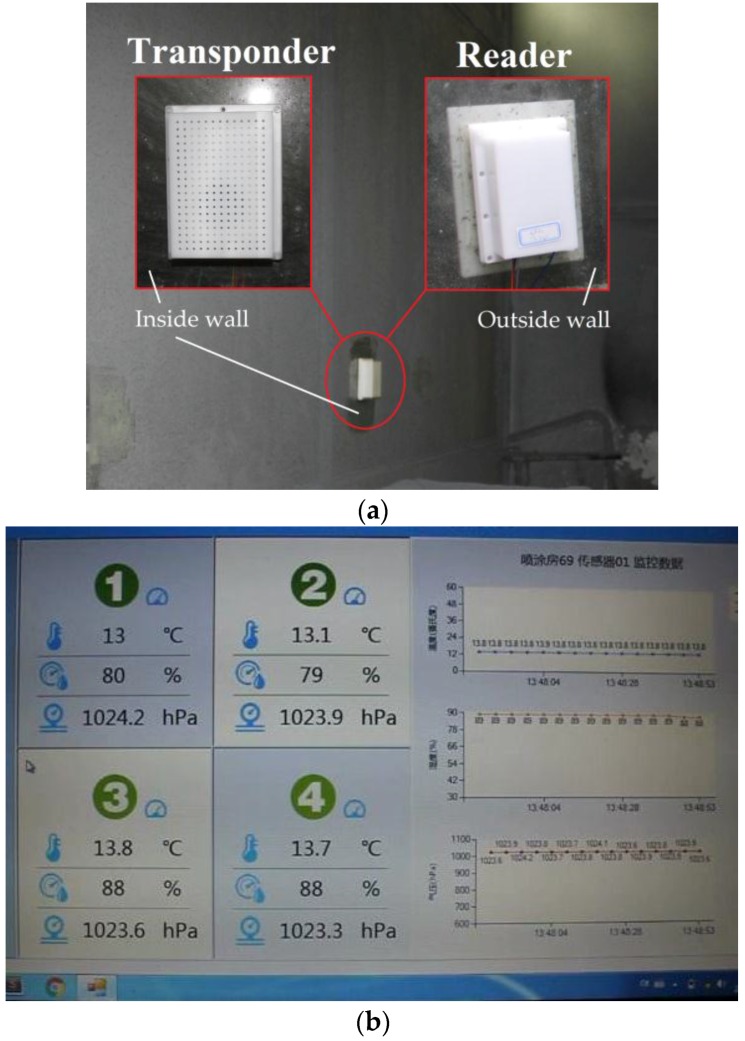
Demonstration of the monitoring systems in spray painting workshop (**a**) Installation of the transponder and reader on the glass wall of the spray painting workshop; (**b**) A screenshot of the monitoring software showing both real-time data and history records.

After the measurement, the passive wireless hermetic environment monitoring system is installed on the glass wall of the spray painting workshop for demonstration. Figure 15a is the photograph of the transponder and reader installed on the glass wall of the spray painting workshop. The one with porous cap inside the glass wall is the transponder, which monitors the environmental parameters of the spray painting workshop, and the one directly facing the transponder outside the glass wall is the reader, which receives data from the transponder and at the same time provides power for it.

Figure 15b is a screenshot of the monitoring software, which could display both real-time data and historical records of the passive wireless monitoring system. Output results of four monitoring systems are shown on the screen at the same time. Among them, No. 1 and No. 2 monitoring systems are installed in one room, and No. 3 and No. 4 are in another room.

## 5. Conclusions

A passive wireless hermetic environment monitoring system was designed, fabricated and demonstrated for the spray painting workshop. The proposed system is composed of a transponder and a reader, and circuit design of each part is given in detail. Because the spray painting workshop contains a large amount of paint mist, the package of the transponder was designed carefully. Three different filter films for the package were compared. The experimental results show that the composite filter film AAO/PTFE has the minimum pollution and the smallest change of water vapor flux. After fabrication, the temperature, humidity and pressure characterizations and the remote sensing distance of the monitoring system were measured. Finally, the passive wireless hermetic environment monitoring system was installed on the glass wall of the spray painting workshop and was successfully demonstrated.
